# Drug Resistant Fetal Arrhythmia in Obstetric Cholestasis

**DOI:** 10.1155/2015/890802

**Published:** 2015-03-03

**Authors:** Nahide Altug, Ayse Kirbas, Korkut Daglar, Ebru Biberoglu, Dilek Uygur, Nuri Danisman

**Affiliations:** ^1^Department of Pediatric Cardiology, Zekai Tahir Burak Women's Health Education and Research Hospital, 06680 Cankaya, Ankara, Turkey; ^2^Department of Perinatology, Zekai Tahir Burak Women's Health Education and Research Hospital, 06680 Cankaya, Ankara, Turkey

## Abstract

Obstetric cholestasis (OC) is a pregnancy specific liver disease characterized by increased levels of bile acid (BA) and pruritus. Raised maternal BA levels could be associated with intrauterine death, fetal distress, and preterm labor and also alter the rate and rhythm of cardiomyocyte contraction and may cause fetal arrhythmic events. We report a case of drug resistant fetal supraventricular tachycardia and concomitant OC. *Conclusion.* If there are maternal OC and concomitant fetal arrhythmia, possibility of the resistance to antiarrhythmic treatment should be kept in mind.

## 1. Introduction

Rhythmic contractions of fetal heart begin on the 21st-22nd days after conception. The conduction system of the fetal heart is physiologically almost mature by the 16th gestation week [1]. Although fetal arrhythmia might be determined as early as the 17th week, it is mostly diagnosed between the 28th and 32nd weeks. Though most of them are generally benign and do not need treatment, fetal supraventricular tachycardia (SVT) may be associated with significant morbidity and mortality, especially when complicated with hydrops. Medical therapy, transplacental or direct fetal, would be used to control the arrhythmia and prevent or reverse fetal hydrops [2].

Obstetric cholestasis (OC) is characterized by pruritus and impaired release of bile from hepatic cells resulting in elevated serum bile acid (BA) levels (>10 mmol/L). The underlying mechanism and pathophysiology of the cholestasis are not clear involving environmental, hormonal, and genetic factors [3–5]. High BA levels, especially those above 40 *μ*mol/L, were found to be associated with higher rates of fetal complications. Fetal demise mostly occurs late in pregnancy possibly related to fetal effects of BAs and cardiac rhythm abnormalities [6–8]. Maternal treatment with ursodeoxycholic acid (UDCA) has been proven to provide significant relief of symptoms, reduce serum biliary acid levels, and prolong pregnancy duration [3, 6].

Fetal atrial flutter and SVT were rarely reported type of arrhythmias in association with OC [9, 10]. Experimental researches have shown a detrimental effect of high biliary acid levels on cardiomyocytes and vasoconstrictive effect on placental chorionic veins resulting in fetal arrhythmia, fetal distress, and asphyxia [11–13]. It is likely that transport of BAs across the plasma membrane can influence their adverse effects on contraction and calcium dynamics and binding to the muscarinic M2 receptor in the neonatal hamster cardiomyocytes [14, 15].

Herein, we present a case of drug resistant fetal SVT and concomitant maternal OC.

## 2. Case Presentation

**Figure 1:**
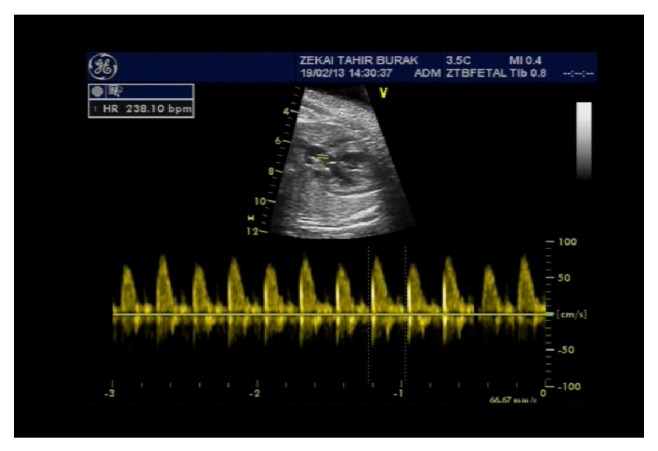
M-mode imaging is demonstrating a heart rate of 238 bpm.

A 21-year-old primigravida was admitted to the clinic at 34 weeks of gestation with generalized pruritus. A diagnosis of OC was made with elevated aspartate aminotransferase (AST), alanine aminotransferase (ALT), and fasting and nonfasting BAs levels (48 U/L, 64 U/L, and 68 and 86 umol/L, resp.). It was noticed that the fetal heart rate was increased on ultrasonography. Fetal echocardiography determined fetal SVT with a heart rate 238/min (Figure 1). There was no evidence of fetal hydrops or heart failure. The fetal cardiac anatomy was unremarkable except for trace tricuspid incompetence. UDCA (15 mg/kg twice a day) was started for OC and oral digoxin (25 mg twice a day) was added as first-line therapy to control the fetal tachyarrhythmia after confirming maternal normal cardiac status. Two doses of 12 mg of betamethasone were given to prevent neonatal respiratory distress syndrome.

Fetal surveillance was assessed with serial ultrasonography, cardiotocography, and fetal echocardiography. The mother was monitored by electrocardiography (ECG). Two days later, even though the maternal digoxin level reached the effective serum dose (0.9 *μ*g/L), the fetus still remained in SVT with mild tricuspid insufficiency. Sotalol treatment (80 mg twice a day) was added to the digoxin therapy. Short SVT runs were observed frequently on the next day and persisted on subsequent days on fetal echocardiography. Weekly fetal ultrasound evaluations showed short SVT episodes with no marked fetal cardiac compromise other than mild tricuspid regurgitation.

Dose of UDCA was increased to 20 mg/kg twice a day 10 days later since the level of AST, ALT, and fasting and nonfasting BA levels showed mild increasing pattern (51 U/L, 68 U/L, and 70 and 88 umol/L, resp.). Her pruritus slowly improved and AST, ALT, and fasting and nonfasting BA levels slowly decreased two weeks later (38 U/L, 47 U/L, and 48 and 55 umol/L, resp.).

Labor induction was discussed with the patient due to nonreassuring fetal status and decreased fetal movements at 37 + 1 weeks of gestation. Oxytocin was started after confirmed fetal lung maturity by using lamellar body count method. The baby whose weight was 2610 grams and APGAR score was 7/9 at the 1st and 5th min was delivered by immediate caesarean section because of intrapartum fetal distress. Meconium-stained amniotic fluid was noted.

The neonate was admitted to the neonatal intensive care unit for cardiac monitoring. ECG showed a regular rhythm with no abnormality. Neonatal echocardiography demonstrated structurally normal heart. Holter ECG showed occasional episodes of SVT runs. The baby presented stable condition with normal sinus rhythm on daily monitorization and was discharged on day 7. He was well with no relapse of SVT at postnatal followup.

## 3. Discussion

SVT is one of the most common forms of fetal tachyarrhythmia characterized on M-mode or Doppler studies by a 1 : 1 ratio of atrial to ventricular contractions [1]. Fetal SVT has wide spectrum of presentation, ranging from an intermittent tachycardia with no hemodynamic compromise to hydrops fetalis and perinatal demise. The presence of fetal hydrops in SVT is associated with a reduction of fetal therapeutic response rates (66% compared with 83% in the nonhydropic fetus) and an increased perinatal mortality [1, 2].

Fetal rhythm abnormalities may occur during the second and the third trimester in OC [9, 10]. Experimental and clinical studies have demonstrated that the fetal heart is affected by BAs [11, 12]. Taurocholate is the principal BA increased in the fetal compartment in OC. It is hypothesized that these BAs are passed from the mother to the fetus; Strehlow et al. demonstrated that taurocholate causes speed and rhythm changes on neonatal rat cardiomyocytes in vivo; therefore, the mechanism of fetal death in OC may be through a fetal cardiac event that is caused by the effect of BAs on the fetal cardiac conduction system [13]. In vitro studies on rat cardiomyocytes have shown that elevated BAs can decrease the contraction rate, reduce the contraction amplitude, prevent cardiomyocyte synchronization, increase loss of cell integrity, and reduce the duration of the action potentials [11–13]. Fan et al. showed that left ventricular global longitudinal strain, systolic strain rate, and diastolic strain rate were decreased significantly in fetuses of cholestatic mothers compared with control fetuses [12]. In fetuses of cholestatic mothers, bradytachycardia and a longer PR interval were observed. Strehlow et al. reported that PR interval was significantly higher in fetuses with OC than in control fetuses [13]. The “pacemaker” function of cardiac myocytes would have been altered by the BA in fetuses.

Gorelik et al. found reduced contraction amplitude due to taurocholate using scanning ion conductance microscopy [14, 15]. This change was reversible after the removal of taurocholate in adults but not in neonatal cardiomyocytes that were exposed to higher concentrations (>0.3 mmol/L). In general, both fetal cardiac systolic and diastolic functions were impaired, especially when associated with serum total BAs greater than 40 *μ*mol/L in mothers with OC.

It has been also demonstrated that high concentrations of BAs induce arrhythmias [16] and may cause electrocardiographic changes in adults [17].

The mechanism of arrhythmia induced by BAs had been described by Abdul Kadir et al. which was associated with a desynchronization of calcium-ion dynamics in cultured neonatal rat cardiomyocytes treated with taurocholate that acts as a partial agonist at the muscarinic M2 receptors [18]. UDCA reduces the total serum BA level and treats pruritus and hepatic dysfunction accompanying OC. The results also show that UDCA is a potential antiarrhythmic agent for treating fetal arrhythmia in OC [19].

Sustained SVT may cause hemodynamic complications by decreasing cardiac output and may develop congestive heart failure and hydrops fetalis and it is an important factor for fetal mortality. Aim of the treatment is to provide sinus rhythm and reverse of cardiac dysfunction. In utero the management approach and the choice of treatment depend on the underlying pathophysiology of the arrhythmia, fetal heart rate and persistence of SVT, gestational age, and institutional experience. All medical agents have the risks of adverse fetal and maternal effects including the risks of maternal arrhythmias. Digoxin is widely accepted as a first-line antiarrhythmic drug (maternal or fetal administration) with a success rate of 80–85% [20]. Generally, treatment is more successful if fetal hydrops is absent. In cases of severe cardiac dysfunction with hydrops the fetal response to maternally administered digoxin is poor. Digoxin has a small therapeutic window and a high incidence of toxicity. Concerns regarding increased mortality in adults with higher serum levels of digoxin have led many clinicians to consider a serum concentration in a lower state [21]. Amiodarone, sotalol, procainamide, adenosine, and flecainide are used usually when digoxin treatment fails to restore a normal sinus rhythm. If there is liver disease, drugs such as amiodarone and flecainide which are metabolized by liver have to be used cautiously or not at all when there is evidence of significant liver dysfunction. Sotalol is a nonselective B-adrenergic blocker agent with class III antiarrhythmic agent [2, 20]. It has been used as a first-line agent in a number of cases in the treatment of fetal SVT with or without hydrops. Its transfer from placenta to the fetus is almost complete. Merriman et al. reported a fetal SVT with worsening cardiac function at 28 weeks. Digoxin therapy was initiated and sotalol was later added for new-onset hydrops. It was concluded that digoxin and sotalol therapy can be successful with resolution of hydrops with a favorable outcome [22]. Sotalol is also highly recommended for the therapy of hydropic fetuses, atrial flutter, or other drug resistant cases [22, 23]. Al Inizi et al. had described that fetal atrial flutter had developed during labour induction of a pregnant woman at 37 weeks, who was diagnosed with OC [9]. Shand et al. also reported a fetal SVT with hydrops and OC which used digoxin and sotalol to treat fetal SVT that was resistant to antiarrhythmic drugs [10]. They discussed that maternal investigations may be appropriate in connection with OC on antiarrhythmic resistant fetal SVTs.

In the present case, no marked cardiac abnormality was determined on fetus of the OC by fetal USG and echocardiography except tachyarrhythmia. Digoxin was started as a first-line medication for SVT. Two days later, sotalol was added to the treatment due to sustained SVT on fetal echocardiography. The mother was administered UDCA in the same period. Many clinicians use UDCA to treat OC despite limited information on its effectiveness in reducing fetal complications, although it has been shown to improve some of the biochemical abnormalities and symptoms of OC [15]. As mentioned before, digoxin is mainly excreted unchanged in the urine and sotalol does not undergo first-pass metabolism by the liver, so these antiarrhythmic agents were preferred as the medical treatment agents. Despite the combined antiarrhythmic drug therapy, fetal SVT could not have been controlled completely. It is possible that the failure of complete response to the antiarrhythmic medication may have been due to the action of the BAs on the fetal myocardium as reported by Shand et al. [10]. There is limited information as to the ideal monitoring protocol or timing of delivery, although many clinicians undertake elective delivery at 37-38 weeks of gestation because of an increased risk of stillbirth after 37 weeks [10, 23].

In conclusion, we have presented a case of fetal SVT with concomitant OC. We suggest that increasing BAs may also cause both fetal tachycardia and resistance to the treatment of arrhythmia. Resistance to antiarrhythmic therapies may be augmented by the myocardial effects of OC and remains a challenge for management. If there are maternal OC and concomitant fetal arrhythmia, possibility of the resistance to antiarrhythmic treatment should be kept in mind.
